# Surgical arrest of post-tonsillectomy haemorrhage: hospital episode statistics 2016–2022

**DOI:** 10.1308/rcsann.2024.0036

**Published:** 2024-05-24

**Authors:** C Heining, M Clark

**Affiliations:** ^1^ University Hospitals Bristol and Weston NHS Foundation Trust, UK; ^2^ Gloucestershire Hospitals NHS Foundation Trust, UK

**Keywords:** Tonsillectomy, Consent, Coblation, COVID

## Abstract

**Introduction:**

Return to theatre for arrest of post-tonsillectomy haemorrhage represents a significant complication of a commonly performed Ear, Nose and Throat procedure. We used Hospital Episode Statistics data to quantify this risk. This method has been used previously for data from 2002–2004 and again for 2010–2016. In this article, coblation tonsillectomy was considered separately as it had not been analysed in previous studies.

**Methods:**

We used Hospital Episode Statistics data provided by the Department of Health to determine the risk of return to theatre for patients undergoing tonsillectomy between 2016 and 2022. Adults and children were analysed separately.

**Results:**

Between 1 April 2016 and 30 April 2022, 179,172 tonsillectomies were performed (not including coblation tonsillectomy), 4,311 (2.41%) of which returned to theatre for control of postoperative bleeding. In children, 1.16% returned to theatre, whereas in adults, 3.80% returned (*p*<0.05). When including coblation tonsillectomy, the return to theatre rate was 0.82% in children, 3.46% in adults and 1.92% overall.

**Conclusions:**

This study shows that adults remain more than three times more likely than children to require a return to theatre for arrest of haemorrhage following tonsillectomy. The rates of post-tonsillectomy haemorrhage decrease when coblation tonsillectomies are added to the analysis. The rate of return to theatre for post-tonsillectomy haemorrhage seems to have stabilised compared with previous work carried out. The authors recommend further work to assess the complication rate of tonsillectomy in the UK and to compare coblation tonsillectomy with other techniques.

## Introduction

Post-tonsillectomy haemorrhage is a significant complication of a commonly performed Ear, Nose and Throat procedure. Return to theatre for arrest of post-tonsillectomy haemorrhage represents the more significant cases of bleeding and, as such, it is important to quantify this risk so patients can be appropriately consented. While collecting data on post-tonsillectomy bleeds managed conservatively is challenging, those that return to theatre are formally recorded and so likely to be a reliable source of data. We used Hospital Episode Statistics (HES) data provided by the Department of Health to determine the risk of return to theatre for patients undergoing tonsillectomy between 2016 and 2022. This method was employed previously for data between 1998 and 2002 and again between 2010 and 2016. It was repeated in this study to observe any changes over this time period.

## Methods

‘Main procedures and interventions’ data for the available years (2016–2022) were downloaded in Microsoft Excel format. The operative (OPCS-4) codes F34.1, F34.2, F34.3, F34.4, F34.8 and F34.9 relating to tonsillectomy procedures and code F36.5 relating to the surgical arrest of post-tonsillectomy bleeding were considered. Codes regarding excision on tonsil remnant (F34.5) and excision of lingual tonsil (F34.6) were excluded from the study. Coblation tonsillectomy (F34.7) was considered separately as it had been excluded from previous studies. The decision to include it this year reflects the increasing number of tonsillectomies done with coblation nationally, and we wished to investigate what effect this might have on bleed rates.

Data for children (0–14 years) and adults (15–75+ years) were compared and assessed statistically with a chi-square test. This was the identical methodology used in two previous audits, where the HES data were available only in these two age categories.^1,2^ Recent HES data are provided in 24 age categories, which were amalgamated to allow for useful comparison.

## Results

Between 1 April 2016 and 30 April 2022, 179,172 tonsillectomies were performed (not including coblation tonsillectomy), 4,311 (2.41%) of which returned to theatre for control of postoperative bleeding. In children, 94,717 tonsillectomies were performed and 1,098 (1.16%) returned to theatre. In adults, 84,455 were performed and 3,213 (3.80%) returned to theatre (Table 1).

**Table 1:** Patients requiring tonsillectomy and theatre for arrest of post-tonsillectomy haemorrhage in England 1 April 2016 to 30 April 2022 (excluding coblation tonsillectomy)

Years	Tonsillectomy, not including coblation (*n*)			Surgical arrest of post-tonsillectomy haemorrhage (*n*)			Return to theatre (%)		
	0–14 years	15–75+ years	Total	0–14 years	15–75+ years	Total	0–14 years	15–75+ years	Total
2016–2017	23,630	18,859	42,489	259	713	972	1.10	3.78	2.29
2017–2018	19,390	16,979	36,369	244	627	871	1.26	3.69	2.39
2018–2019	21,071	17,358	38,429	219	672	891	1.04	3.87	2.32
2019–2020	16,169	14,874	31,043	217	613	830	1.34	4.12	2.67
2020–2021	5,422	6,122	11,544	62	209	271	1.14	3.41	2.35
2021–2022	9,035	10,263	19,298	97	379	476	1.07	3.69	2.47
Totals	94,717	84,455	179,172	1,098	3,213	4,311	1.16	3.80	2.41
2018 study	150,900	116,259	267,159	1,375	3,661	5,027	0.91	3.15	1.88
2004 Study	131,577	88,920	220,497	500	1,304	1,804	0.38	1.47	0.82

**Figure 1:**
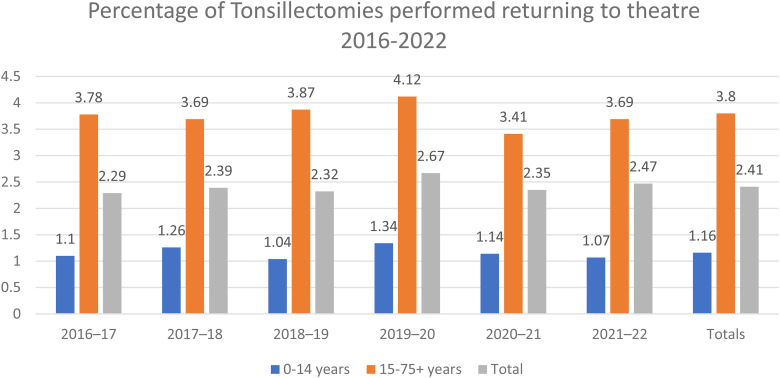
Percentage of noncoblation tonsillectomies performed returning to theatre from 2016 to 2022

**Figure 2:**
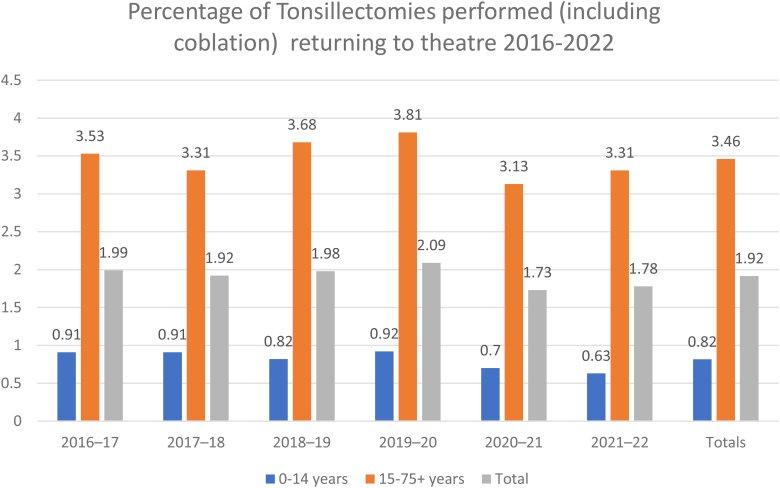
Percentage of all tonsillectomies performed (including coblation) returning to theatre from 2016 to 2022

The difference between the incidence of adults and children returning to theatre was statistically significant (*p*<0.05). This is a continuation of results from the previous two audits. The average number of tonsillectomies performed per year has fallen by 32.9% since the previous study (Table 2). Some of this may reflect change in clinical practice, but the number of tonsillectomies particularly fell from 2020 onwards, likely reflecting the effects of the COVID-19 pandemic. Comparisons made with the previous studies demonstrated an overall increase in return to theatre rates from 0.82% in 2004 to 1.88% in the 2018 study to 2.41% from our data. The pattern is replicated in both children (0.38% to 0.91% to 1.16%) and adult (1.47% to 3.15% to 3.80%) subgroups Figures 1 and 2.

**Table 2:** Total tonsillectomies performed during each year of this and previous comparison studies

Year	Tonsillectomies (*n*)	
Clark and Waddell^1^		
1998–1999	67,139	
1999–2000	59,117	
2000–2001	46,001	
2001–2002	48,321	
Average	55,145	
Osborne and Clark^2^		
2010/2011	43,717	
2011/2012	42,726	
2012/2013	44,071	
2013/2014	46,644	
2014/2015	46,137	
2015/2016	43,864	
Average	44,527	
Heining and Clark (this study)	Without coblation	Totals including coblation (tonsillectomies by coblation)
2016–2017	42,489	48,793 (6,304)
2017–2018	36,369	43,673 (7,304)
2018–2019	38,429	47,039 (8,610)
2019–2020	31,043	39,778 (8,735)
2020–2021	11,544	15,546 (4,002)
2021–2022	19,298	26,809 (7,511)
Average	29,862	36,940 (7,078)

## Discussion and conclusions

This study shows that adults remain more than three times more likely than children to require a return to theatre for arrest of haemorrhage following tonsillectomy. The overall return to theatre rate has increased by 0.53% since 2016.^2^ It is interesting to note that the rates of post-tonsillectomy haemorrhage decrease when coblation tonsillectomies are added to the analysis (Table 3). In fact, when coblation tonsillectomies are added to the analysis, the rate of post-tonsillectomy return to theatre is similar to the previous audit published in 2018. The rate of post-tonsillectomy haemorrhage has reduced in children when coblation tonsillectomies are considered.

**Table 3:** Patients requiring tonsillectomy and theatre for arrest of post-tonsillectomy haemorrhage in England 1 April 2016 to 30 April 2022 including coblation tonsillectomy

Years	Tonsillectomy, including coblation (*n*)			Surgical arrest of post-tonsillectomy haemorrhage (*n*)			Return to theatre (%)		
	0–14 years	15–75+ years	Total	0–14 years	15–75+ years	Total	0–14 years	15–75+ years	Total
2016–2017	28,584	20,203	48,793	259	713	972	0.91	3.53	1.99
2017–2018	26,794	18,922	43,673	244	627	871	0.91	3.31	1.92
2018–2019	26,689	18,279	47,039	219	672	891	0.82	3.68	1.98
2019–2020	23,683	16,081	39,778	217	613	830	0.92	3.81	2.09
2020–2021	8,858	6,675	15,546	62	209	271	0.70	3.13	1.73
2021–2022	15,320	11,467	26,809	97	379	476	0.63	3.31	1.78
Totals	129,928	91,627	221,638	1,098	3,213	4,311	0.82	3.46	1.92
2018 study	150,900	116,259	267,159	1,375	3,661	5,027	0.91	3.15	1.88
2004 Study	131,577	88,920	220,497	500	1,304	1,804	0.38	1.47	0.82

Unfortunately, the data captured by HES do not differentiate between extracapsular coblation tonsillectomy and intracapsular coblation tonsillotomy. It is acknowledged many advocates of coblation use intracapsular in preference to extracapsular techniques.^3^ However, the authors are unable to comment on the relative merits or drawbacks of these techniques based on the data currently collected by HES.

Coblation is a more recently developed technique for performing tonsillectomy and its possible benefits over more traditional techniques remain unclear.^4,5^ Despite equivocal evidence of benefit, it is increasing in popularity—for example, only 2,437 coblation tonsillectomies were coded in the year 2010–2011,^6^ which compares with an average of over 7,000 per year for our study period. Our study period includes the period from 2020 onwards where general operating numbers would have been affected by the COVID-19 pandemic. The numbers done by coblation would possibly be higher still if not for the effects of the pandemic. The authors felt the increasing popularity of the coblation technique needs to be considered when analysing data from recent years. Ignoring coblation tonsillectomy would ignore a significant proportion of tonsillectomies being performed and would artificially increase the rate of return to theatre.

It is impossible to tell from the available data whether the decreased rate of return to theatre is down to reduced complications from coblation compared with other techniques, or if it is simply down to increasing the total number tonsillectomies analysed. Further high-quality work comparing coblation tonsillectomy with other techniques is recommended.

HES also does not collect data on technique of tonsillectomy, other than coblation. Therefore, the authors are unable to comment on how bipolar dissection may compare with cold steel tonsillectomy, or any other techniques. The authors are also unable to comment on how the popularity of each of these techniques has changed over the study period. Further high-quality prospective work comparing a range of tonsillectomy techniques is recommended.

The other major confounder that makes analysis across the timespans challenging is the effect of the COVID-19 pandemic. COVID-19 resulted in a reduction or complete halt of elective services (such as tonsillectomy)^7^ across many UK hospitals for a period of time. As such, the cohort of patients undergoing tonsillectomy are likely to have been different from previously analysed time periods. There are more likely to have been a greater number of diagnostic tonsillectomies for histology.

Children undergoing tonsillectomy for obstructive sleep apnoea (OSA) are likely to have had more severe OSA or other medical problems necessitating the expedition of their treatment. Patients undergoing tonsillectomy for recurrent tonsillitis will possibly have had more episodes than previously. More complex patients, such as these, are perhaps more likely to have complications than more straightforward cases. However, the rate of return to theatre in the years 2020–2021 and 2021–2022 are actually slightly lower than the average return-to-theatre rate for the study period.

There has also been a further reduction in the total number of tonsillectomies performed. This will again be affected by the COVID-19 pandemic, but also by the ongoing effect of the Scottish Intercollegiate Guideline Network guidelines on eligibility for tonsillectomy.^8^ As previous studies have discussed,^1,2^ the decline in the overall number of tonsillectomies performed may mean less surgical experience of tonsillectomies for middle grade surgeons and thus less experience of managing their complications.

The authors note HES data are based upon clinical coding of procedures and interventions; improvements or changes in data reporting methods may partially explain some of the changes measured above. It would seem the rate of return to theatre for post-tonsillectomy haemorrhage has stabilised compared with previous audit. It is uncertain whether this reflects a true stabilisation of the complication rate, or simply the change in tonsillectomy numbers brought about by the COVID-19 pandemic. The increased use of coblation techniques may also be relevant. The authors recommend further work to assess the complication rate of tonsillectomy in the UK and to compare coblation tonsillectomy with other techniques.
